# Perceived Injustice After Mild Traumatic Brain Injury

**DOI:** 10.1097/HTR.0000000000000698

**Published:** 2021-06-15

**Authors:** Kaisa Mäki, Taina Nybo, Marja Hietanen, Antti Huovinen, Ivan Marinkovic, Harri Isokuortti, Susanna Melkas

**Affiliations:** Neuropsychology (Ms Mäki and Drs Nybo and Hietanen) and Neurology (Drs Huovinen, Marinkovic, Isokuortti, and Melkas), University of Helsinki and Helsinki University Hospital, Helsinki, Finland.

**Keywords:** mild traumatic brain injury, perceived injustice, postconcussion symptoms, return to work

## Abstract

To examine perceived injustice and its associations with self-reported symptoms and return to work at 3 months after injury in a prospectively recruited sample of patients with mild traumatic brain injury (mTBI).

**Design:**

Observational study.

**Setting:**

TBI outpatient unit.

**Participants:**

Adult patients aged 18 to 68 years with mTBI (*n* = 100) or orthopedic injury ([OI]; *n* = 34).

**Main Measures:**

The Injustice Experience Questionnaire (IEQ) and its associations with the Rivermead Post Concussion Questionnaire (RPQ), Beck Depression Inventory–Second Edition (BDI-II), PTSD Checklist–Civilian Version (PCL-C), and Pain Visual Analog Scale (PVAS). Information on injury-related characteristics, compensation seeking and litigation, and return-to-work status was also collected.

**Results:**

Median IEQ total score was 3 (range, 0-23) in the mTBI group and 2.5 (range, 0-25) in the OI group. In the mTBI group, IEQ was significantly correlated with RPQ (*r*_s_ = 0.638, *P* < .01), BDI-II (*r*_s_ = 0.612, *P* < .01), PCL-C (*r*_s_ = 0.679, *P* < .01), and PVAS (*r*_s_ = 0.232, *P* < .05). The association between IEQ and PCL-C (*r*_s_ =0.797, *P* < .01) and BDI-II (*r*_s_ = 0.395, *P* < .05) was also found in the OI group. In both groups, patients who were still on sick leave at 3 months after injury tended to report higher perceived injustice (IEQ total score) than patients who had returned to work or studies. However, this difference did not reach statistical significance.

**Conclusions:**

Perceived injustice is associated with self-reported symptoms in patients with mTBI. Our results suggest that perceived injustice could be a relevant construct to consider in clinical management of patients with mTBI. Also, perceived injustice could be a potential target for psychological interventions promoting recovery after mTBI.

PERCEIVED INJUSTICE is a cognitive appraisal process characterized by a tendency to see one's situation unfair, one's losses severe and irreparable, and to attribute blame to others for one's suffering.^1^

Previous studies show that high levels of perceived injustice are a risk factor for pain, disability, and psychological distress in individuals with traumatic musculoskeletal injury and chronic pain conditions.^2^ Also, it has been suggested that perceived injustice might be associated with a decreased likelihood of return to work after traumatic musculoskeletal injury.^1^,^3^–^5^

Recovery expectations have been proposed as one potential mechanism through which perceived injustice might impact symptom experience and outcome after injury.^4^,^6^ It is known that individuals preferentially process information that is consistent with expectancies and discount information that is inconsistent with expectancies.^7^ It has been suggested that low expectancies of recovery and focus on expectancy consistent information might reduce effort and motivation to engage in behaviors that promote recovery, such a adhering to rehabilitation.^4^,^6^ Attributing blame to others for one's negative circumstances (external blame attribution) could be another factor that might promote passive orientation and thus compromise optimal recovery potential.^8^ There is evidence that external blame attribution might be a risk factor for worse emotional distress among patients with moderate or severe TBI.^9^ It is also noteworthy that perceptions of injustice not only are cognitive constructs but can also in some cases stem from a reality that is characterized by justice violations.^8^ Litigation and compensation issues are known to be associated with adverse recovery outcomes after injury.^2^,^10^–^12^ It has been proposed that injustice perceptions and retribution motives could have role in this association.^1^,^8^

There is some preliminary evidence suggesting that perceived injustice might be associated with symptom experience after mild traumatic brain injury (mTBI). Iverson and colleagues^13^ used the Injustice Experience Questionnaire (IEQ)^1^ to assess injustice perceptions of 102 adult patients (aged 19-64 years) who had been referred to specialty concussion clinics because of prolonged symptoms on an average 12 weeks after mTBI. They found that levels of perceived injustice were similar to those previously reported in patients with chronic musculoskeletal injury and chronic pain.^13^ Also, they found that higher perceived injustice correlated with more postconcussion symptoms, depressive symptoms, traumatic stress, and pain.^13^

To date, perceived injustice after mTBI has been studied only in patients who are seeking treatment of prolonged symptoms.^13^ It is not known whether previous results are generalizable to the less selected population of patients presenting for routine mTBI evaluation. Also, the association between perceived injustice and return to work has not been previously studied in patients with mTBI.

The aim of the present study was to examine perceived injustice and its associations with self-reported symptoms and return to work at 3 months after injury in a prospectively recruited unselected cohort of patients with mTBI.

## MATERIALS AND METHODS

The study protocol was approved by the Helsinki University Hospital Ethics Committee of Medicine. All participants provided written informed consent according to the Declaration of Helsinki.

### Definition of mTBI

We used the World Health Organization (WHO) Collaborating Centre Task Force on Mild Traumatic Brain Injury criteria for mTBI.^14^ The criteria include (i) 1 or more of the following: confusion or disorientation, loss of consciousness for 30 minutes or less, posttraumatic amnesia less than 24 hours, and/or other transient neurological abnormalities such as focal signs, seizure, and intracranial lesion not requiring surgery; and (ii) Glasgow Coma Scale score of 13 to 15 after 30 minutes or later upon presentation for healthcare.^14^ As suggested by Williams and colleagues,^15^ we use the term “complicated mTBI” to refer to patients with trauma-related lesions in neuroimaging.

### Participants

The study cohort consisted of 131 adult patients with mTBI from the Traumatic Brain Injury Outpatient Clinic of Helsinki University Hospital. Patients were referred to this unit from emergency departments and primary healthcare services as a part of routine mTBI management, not because of specific symptoms or compensation issues. All patients met the criteria for mTBI^14^ and were enrolled to the study within 12 days or less after sustaining injury. Exclusion criteria for this study were age less than 18 years or more than 68 years, history of diagnosis of severe psychiatric disorder (schizophrenia, schizoaffective disorder), developmental disability, visual or hearing impairment, and contraindication for magnetic resonance imaging (MRI). In addition, patients with alcohol and/or drug dependence were excluded, as this condition could significantly hamper the patient's commitment to examinations and follow-up. Dependence was defined according to the *Diagnostic and Statistical Manual of Mental Disorders, 4th edition* (*DSM-IV*) criteria.^16^

The orthopedic injury (OI) control group consisted of 40 adult patients treated in the Trauma Emergency Department of Helsinki University Hospital because of lower extremity OI (ankle fracture). Consecutive patients with OI were approached after they had been released from the emergency unit. Patients with OI were excluded from the study if there was any suspicion of having sustained a head injury based on hospital records and patient interview. Otherwise, the inclusion and exclusion criteria for the OI group were as described previously.

Of the original study cohort, 31 (23.6%) patients with mTBI and 6 (15%) patients with OI had missing data in the self-report measures used in the present study. Thus, the final study sample consisted of 100 patients with mTBI and 34 patients with OI.

### Procedure

Patients with mTBI underwent brain structural MRI (3T, Magnetom Verio; Siemens, Erlagen, Germany) 3 to 36 (median 10) days after injury. In line with normal clinical practice, the patients were informed about their MRI results by a neurologist. Findings and their implications were discussed, and the scans were shown to the patient if he or she so wished. Information on injury-related characteristics was collected from hospital records. Information on compensation seeking and litigation status was based on self-report and collected in a structured interview 11 to 53 (median 31) days after injury. Perceived injustice and symptom experience were assessed with self-report measures as part of a more comprehensive neuropsychological assessment 64 to 149 (median 88) days after mTBI. Self-reported return-to-work status was collected as part of the neuropsychological assessment, and ongoing sick leave due to injury was then verified from the electronic patient records. Patients with OI completed all the same procedures as patients with mTBI, as soon after recruitment as was convenient for them.

### Measures

#### Injustice Experience Questionnaire^1^,^17^

The IEQ is a self-report measure of injury-related perceived injustice. The IEQ consists of 12 statements assessing the responder's appraisals of their injury and its consequences in terms of unfairness (eg, “It all seems so unfair”), irreparability of loss (“My life will never be the same”), and attribution of blame (“I am suffering because of someone else's negligence”). Responders rate how often they experience each of the 12 injustice-related thoughts on a 5-point scale from 0 to 4 as follows: never (0), rarely (1), sometimes (2), often (3), all the time (4). A total score is calculated by adding all items (theoretical range, 0-48). In addition, the proportion of participants endorsing each individual item is calculated. Endorsement is defined as a rating of “sometimes” or greater (≥2).

#### Rivermead Post Concussion Questionnaire^18^

The Rivermead Post Concussion Questionnaire (RPQ) is a 16-item self-report measure of somatic, emotional, and cognitive complaints that are commonly reported after mTBI. Responders rate the presence and severity of symptoms over the past 24 hours relative to their experience of the same symptoms prior to injury on a 5-point scale from 0 to 4 as follows: not experienced the symptom (0), no more of a problem (1), a mild problem (2), a moderate problem (3), and a severe problem (4). A total score is calculated by adding all items with a score greater than 1 (theoretical range, 0-64).

#### Beck Depression Inventory–Second Edition^19^

The Beck Depression Inventory–Second Edition (BDI-II) is a 21-item measure assessing symptoms of depression. Each item consists of 4 alternative statements. Responders are asked to endorse the one that best describes how they are currently feeling. Items are scored on a scale from 0 to 3, with higher scores reflecting greater symptom severity. A total score is calculated by adding all items (theoretical range, 0-63).

#### PTSD Checklist–Civilian Version^20^

The PTSD Checklist–Civilian Version (PCL-C) is a self-report measure of posttraumatic stress comprising 17 items that correspond to the key symptoms of PTSD based on the *DSM-IV* criteria.^16^ Responders rate each item on a 5-point scale as follows: not at all (1), a little bit (2), moderately (3), quite a bit (4), and extremely (5). A total score is calculated by adding all items (theoretical range, 17-85).

#### Pain Visual Analog Scale^21^

Pain intensity was assessed with the Pain Visual Analog Scale (PVAS), a commonly used brief measure of pain. The PVAS consists of a 10-cm-long straight line with endpoints defining extreme limits to pain intensity (from “no pain” to “maximum pain ever experienced”). Responders are asked to indicate their current level of pain by placing a mark along the line. Score was determined by measuring the distance (millimeters) on the line between the “no pain” endpoint and the responder's mark (theoretical range, 0-100).

### Statistical analysis

Continuous variables are presented as mean (SD) if normally distributed and as median (range) if not normally distributed. Categorical variables are given as frequencies and percentages. Correlations were calculated using Spearman's rank correlations. For group comparisons, Pearson's chi-square test and Fisher's exact test were used for binary categorical variables, Student's *t* tests for normally distributed continuous variables, and nonparametric Mann-Whitney *U* tests for nonnormally distributed continuous variables. Effect size (*r*) was calculated for statistically significant Mann-Whitney *U*-tests. The level of statistical significance was set at *P* < .05. Statistical analyses were carried out using IBM SPSS Statistics for Windows (version 25; IBM Corp, Armonk, New York).

## RESULTS

### Sample characteristics

The mTBI and OI groups did not differ in terms of age, gender, education, or preinjury employment status (see Table 1). The median time from injury to neuropsychological assessment was significantly longer for the patients with OI than for the patients with mTBI (112 and 88 days, respectively, Mann-Whitney *U* = 2445.0, *P* < .001). Ground-level fall was the most common injury mechanism in both groups. The mTBI group included patients who had been injured in motor vehicle accidents (*n* = 7; 7%), in pedestrian traffic accidents (*n* = 2; 2%), and in violence-related incidences (*n* = 3; 3%), while the aforementioned injury mechanisms were not present in the OI group. Having sustained a work-related injury and seeking compensation were as common in both groups. Few patients were involved in litigation related to their injury (3 patients in the mTBI group, none in the OI group). Forty-two percent of the patients with mTBI had traumatic intracranial lesions on MRI, that is, complicated mTBI.

**TABLE 1:** Descriptive characteristics of patients with mild traumatic brain injury and those with orthopedic injury

Variable	mTBI (*n* = 100)	OI (*n* = 34)	*P* a	Effect size (*r*)
Age, mean (SD), y	40.4 (13.2)	42.4 (11.8)	.437	
Gender (female), *n* (%)	50 (50)	17 (50)	1.000	
Education, mean (SD), y	15.8 (3.6)	15.9 (3.4)	.547	
Working/full-time student prior to injury, *n* (%)	91 (91)	33 (97.1)	.451	
Time since injury at neuropsychological assessment, Md (range), d	88 (64-149)	112 (42-178)	<.001	−0.319
Traumatic intracranial lesions on MRI, *n* (%)	42 (42)	0 (0)		
Cause of injury, *n* (%)				
Motor vehicle accident	7 (7)	0 (0)		
Pedestrian traffic accident	2 (2)	0 (0)		
Bicycle accident	24 (24)	2 (5.9)		
Ground-level fall	29 (29)	16 (47.1)		
Fall from heights	21 (21)	7 (20.6)		
Sports	11 (11)	8 (23.5)		
Violence related	3 (3)	0 (0)		
Other	2 (2)	0 (0)		
Unknown	1 (1)	1 (2.9)		
Work-related injury, *n* (%)	24 (24)	7 (21)	.815	
Compensation seeking, *n* (%)	68 (68)	22^b^ (69)	.937	
Litigation, *n* (%)	3 (3)	0^b^ (0)		

Abbreviations: Md, median; MRI, magnetic resonance imaging; mTBI, mild traumatic brain injury; OI, orthopedic injury.

^a^Categorical variables were compared with Pearson's chi-square tests and Fisher's exact test and continuous variables with *t* tests or Mann-Whitney *U* tests.

^b^Two cases missing, *n* = 32.

As shown in Table 2, patients with mTBI reported more postconcussion symptoms than patients with OI (Mann-Whitney *U* = 1252.0, *P* = .014). The 2 groups did not differ significantly in terms of depressive symptoms, posttraumatic stress symptoms, or pain intensity.

**TABLE 2:** Self-reported symptoms in patients with mild traumatic injury and those with orthopedic injury

	mTBI (*n* = 100), Md (range)	OI (*n* = 34), Md (range)	*P* a	Effect size (*r*)
Postconcussion symptoms (RPQ)	2 (0-32)	0 (0-12)	.014	−0.211
Depressive symptoms (BDI-II)	3 (0-25)	3 (0-18)	.647	
Posttraumatic stress symptoms (PCL-C)	21 (17-54)^b^	19.5 (17-40)	.230	
Pain (PVAS)	3 (0-76)	6 (0-58)	.264	

Abbreviations: BDI-II, Beck Depression Inventory–Second Edition; Md, median; mTBI, mild traumatic brain injury; OI, orthopedic injury; PCL-C, PTSD Checklist–Civilian Version; PVAS, Pain Visual Analog Scale; RPQ, Rivermead Post Concussion Questionnaire.

^a^*P* values are for Mann-Whitney *U* test.

^b^One case missing, *n* = 99.

### Perceived injustice

Median IEQ total score was 3 (range, 0-23) for the mTBI group and 2.5 (range, 0-25) for the OI group. In the mTBI group, most commonly endorsed individual items of the IEQ were as follows: (1) feeling other people don't understand the severity of one's condition (23%); (6) feeling one might be permanently affected (23%); and (5) wanting one's life back (13%). There were no significant differences between the mTBI and OI groups in the IEQ total scores or frequency of endorsing any of the individual items (see Table 3).

**TABLE 3:** Injustice Experience Questionnaire (IEQ) total score and individual item endorsement^a^ in patients with mild traumatic brain injury and those with orthopedic injury

Variable	mTBI (*n* = 100)	OI (*n* = 34)	*P* b
IEQ total score, Md (range)	3 (0-23)	2.5 (0-25)	.907
IEQ individual item endorsement, *n* (%)			
1. Most people don't understand how severe my condition is	23 (23)	9 (26.5)	.651
2. My life will never be the same	11 (11)	7 (20.6)	.242
3. I am suffering because of someone else's negligence	11 (11)	3 (8.8)	.765
4. No one should have to live this way	3 (3)	2 (5.9)	.601
5. I just want my life back	13 (13)	5 (14.7)	.777
6. I feel that this has affected me in a permanent way	23 (23)	7 (20.6)	1.000
7. It all seems so unfair	9 (9)	3 (8.8)	.513
8. I worry that my condition is not being taken seriously	4 (4)	3 (8.8)	.247
9. Nothing will ever make up for all that I have gone through	1 (1)	1 (2.9)	.445
10. I feel as if I have been robbed of something very precious	4 (4)	0 (0)	.305
11. I am troubled by fears that I may never achieve my dreams	12 (12)	3 (8.8)	.440
12. I can't believe this has happened to me	12 (12)	3 (8.8)	.760

Abbreviations: IEQ, Injustice Experience Questionnaire, Md, median; mTBI, mild traumatic brain injury; OI, orthopedic injury.

^a^Endorsement is defined as a rating of 2 or more.

^b^*P* values are for the Mann-Whitney *U* test and Fisher's exact test.

In the mTBI group, perceived injustice was significantly correlated with postconcussion symptoms (*r*_s_ = 0.638, *P* < .01), depressive symptoms (*r*_s_ = 0.612, *P* < .01), posttraumatic stress symptoms (*r*_s_ = 0.679, *P* < .01), and pain intensity (*r*_s_ = 0.232, *P* < .05) (see Table 4). The association between perceived injustice and posttraumatic stress symptoms (*r*_s_ = 0.797, *P* < .01) and depressive symptoms (*r*_s_ = 0.395, *P* < .05) was also found in the OI group (see Table 4).

**TABLE 4:** Spearman correlations between perceived injustice and self-reported symptoms in patients with mild traumatic brain injury and those with orthopedic injury

	mTBI (*n* = 100)	OI (*n* = 34)
Postconcussion symptoms (RPQ)	0.638^a^	0.161
Depressive symptoms (BDI-II)	0.612^a^	0.395^b^
Posttraumatic stress symptoms (PCL-C)	0.679^a^,^c^	0.797^a^
Pain (PVAS)	0.232^b^	0.335

Abbreviations: BDI-II, Beck Depression Inventory–Second Edition; mTBI, mild traumatic brain injury; OI, orthopedic injury; PCL-C, PTSD Checklist–Civilian Version; PVAS, Pain Visual Analog Scale; RPQ, Rivermead Post Concussion Questionnaire.

^a^*P* < .01.

^b^*P* < .05.

^c^One case missing, *n* = 99.

Perceived injustice was not associated with age, gender, education, or time since injury in neither the mTBI group nor the OI group. Having sustained a work-related injury or seeking compensation was not associated with perceived injustice in neither of the 2 groups. To explore the association between injury type and perceived injustice, patients with mTBI were dichotomized into 2 groups based on whether they had been involved in a traffic accident (motor vehicle or pedestrian, *n* = 9) or not. Patients injured in traffic accidents reported higher perceived injustice than other patients with mTBI (Mann-Whitney *U* = 241.0, *P* = .039, *r* = −0.237).

To further explore the injustice experience after mTBI, we evaluated patients with complicated and uncomplicated injuries separately. Patients with complicated mTBI (*n* = 42) reported higher injustice (IEQ total score) than those with uncomplicated mTBI (Mann-Whitney *U* = 1646.5, *P* = .002, *r* = −0.304). Median IEQ total score was 4 (range, 0-23) for the complicated and 1 (range, 0-19) for the uncomplicated mTBI group. In terms of individual IEQ item scores, patients with complicated mTBI endorsed item (2) “My life will never be the same” slightly more often than patients with uncomplicated mTBI (Fisher exact *P* = .049). Other significant differences between the complicated and uncomplicated mTBI groups in any other individual IEQ item endorsement were not detected.

Correlations between perceived injustice, postconcussion symptoms, depressive symptoms, and posttraumatic stress symptoms remained unchanged when analyzed separately for complicated and uncomplicated mTBIs. Association between perceived injustice and pain intensity was found only for the uncomplicated mTBI group (*r*_s_ = 0.47, *P* < .01).

### Return to work

Of the 91 patients with mTBI and 33 with OI who were working or studying full-time prior to their injury, 85 (93.4%) and 28 (84.8%) patients had returned to these activities within the study period, respectively. In the mTBI group, median IEQ total score was 8.5 (range, 0-21) for patients who were still on sick leave and 2 (range, 0-19) for those who had already returned to work or studies. In the OI group, corresponding median IEQ scores were 9 (range, 0-15) and 2 (range, 0-19), respectively. These differences did not reach statistical significance in either group. Explorative analyses of the individual IEQ item endorsement revealed that patients with mTBI who were still on sick leave endorsed item (5) “I just want my life back” more often than those who had returned to work or studies (Pearson χ^2^ = 8.688, *P* = .022). In addition, patients with OI who were still on sick leave endorsed items (1) “Most people don't understand how severe my condition is” and (5) “I just want my life back” more often than those who had returned to work or studies (Pearson χ^2^ = 6.048, *P* = .031, and χ^2^ = 7.235, *P* = .029, respectively). Of the 6 patients with mTBI who were still on sick leave, 2 had uncomplicated TBI and 4 complicated mTBI.

## DISCUSSION

The present study examined perceived injustice and its association with self-reported symptoms at 3 months after mTBI. Perceived injustice was generally low in our cohort. However, perceived injustice was associated with postconcussion symptoms, depressive symptoms, posttraumatic stress symptoms, and pain intensity.

In the present study, ratings of perceived injustice (IEQ total scores) were markedly lower than those previously reported for patients with mTBI^13^,^22^ or musculoskeletal injury.^2^ This is likely explained by the fact that the participants of this cohort were prospectively recruited within 12 days after injury, whereas most previous studies have included only treatment-seeking individuals recruited later in the chronic phase after injury. Indeed, the 3 months postinjury ratings of perceived injustice in the current study are in broad agreement with 3 months postinjury ratings previously reported for patients with whiplash injury prospectively recruited from a primary care setting.^23^

Perceived injustice construct includes aspects of unfairness, severity and irreparability of loss, and external blame attribution.^1^ In the present cohort, the most commonly endorsed IEQ items related to the severity/irreparability aspects of the construct. Intentional injury (violence) and other injuries where other person involvement is common (eg, traffic accidents) were rare in our cohort, which might have impacted endorsement of external blame attribution–related items.

In accordance with Iverson and colleagues,^13^ higher perceived injustice was associated with higher postconcussion symptoms in the mTBI group. Consistent with previous research in patients with mTBI^13^ and chronic pain after musculoskeletal injury,^1^,^17^,^24^–^26^ higher perceived injustice was found to be associated with higher depressive and posttraumatic stress symptoms in both study groups. Most previous studies have shown an association between perceived injustice and pain intensity,^2^,^13^ although conflicting results have also been reported.^17^,^24^,^27^ In the present study, association between perceived injustice and pain intensity was found for the mTBI group but not for the OI group. When patients with uncomplicated and complicated mTBIs were evaluated separately, association between pain and perceived injustice was found only for the uncomplicated mTBI group.

Unlike Iverson and colleagues,^13^ we did not find correlation between compensation seeking and perceived injustice. Seeking compensation from personal insurance or through the Finnish full compensation system applicable for traffic accidents involving motor vehicle and work-related injuries was relatively common in the present study cohort. However, it should be noted that, in the region where this study was conducted, all injured patients are entitled to compensation for lost income due to incapacity for work also through the governmental social benefits scheme. In addition, the majority of patients in this study had already recovered well by 3 months after injury. It could be speculated that both these factors could affect individual's appraisal of his or her injury and reduce the gravity and psychological stress associated with claims process,^28^ making perceptions of injustice less likely to arise. Even so, we found that victims of motor vehicle or pedestrian traffic accidents, one specific type of compensable injury, reported higher perceived injustice than patients whose injury was not traffic related. This finding is in agreement with previous reports,^13^,^29^ but its broader interpretation in the current cohort is difficult as this injury mechanism was rare in our cohort (*n* = 9).

In the current study, patients with complicated mTBI reported higher perceived injustice than patients with uncomplicated mTBI. Prior evidence is mixed on whether patients with complicated mTBI have worse short- and medium-term outcomes than patients with uncomplicated mTBI.^30^–^32^ Thus, this result could be explained by a response to more severe injury associated with more disability. However, it is also possible that knowing that injury had caused a visible lesion might impact patient's appraisal of injury severity and subjective recovery expectations,^4^,^6^,^33^ making perceptions of injustice and irreparability of loss more likely to arise.

It has been suggested that perceived injustice may be associated with a decreased likelihood of return to work in patients with traumatic musculoskeletal injury.^1^,^3^–^5^ This association has not been previously studied in patients with mTBI. In the present study, patients who were still on sick leave did not report significantly higher levels of perceived injustice (IEQ total score) than those who had returned to work or studies within the study period. Considering the extremely small number of patients in the sick leave group, this should be interpreted with caution and further study with larger cohorts is warranted.

It has been suggested that perceived injustice might reduce effort and motivation to engage in behaviors that promote recovery, such a adhering to rehabilitation.^4^,^8^ Thus, psychological interventions taking perceived injustice experience into consideration could provide tools that help patient to shift his or her cognitive focus toward more active agency and thus promote recovery after mTBI. However, further studies are still needed.

The strengths of this study include mTBI cohort prospectively recruited from an unselected population with few exclusion criteria, contributing to the generalizability of the findings. Also, inclusion of the OI comparison group enabled us to control for factors that can affect clinical presentation in patients with mTBI, such as nonspecific effects of traumatic injury and behavioral factors that predispose a person to injury.^34^,^35^

However, there are also some limitations. Despite the routine TBI management practice, all patients with mTBI treated in the local emergency departments and primary healthcare services are not referred to our outpatient clinic. This could have skewed our cohort in favor of those with more complaints. However, considering the low symptom reporting observed, it is unlikely this has caused major bias. Correlational design of the present study precludes any conclusions about directionality or causality. Also, the potential development of perceived injustice over time could not be assessed. The present cohort included patients with mTBI who were involved in litigation. This could have caused bias, as it is known that litigating patients can respond differently on self-report symptoms measures as those who are not involved in litigation.^10^–^12^ However, the number of litigating patients was small (*n* = 3). Removing litigating patients from the analyses did not change the results in any significant way. Time interval between injury and neuropsychological assessment was longer for the patients with OI than for the patients with mTBI, which could have caused bias. Finally, we cannot completely rule out the possibility of covert brain injury in the OI group, although availability of hospital records and careful patient interview should have minimized this possibility.

In conclusion, our results suggest that perceived injustice could be a relevant construct to consider in clinical management of patients with mTBI, especially those reporting persisting symptoms. In the future, development of psychological interventions that target perceived injustice might also provide one potential path to promote recovery after mTBI. However, further research is warranted.
